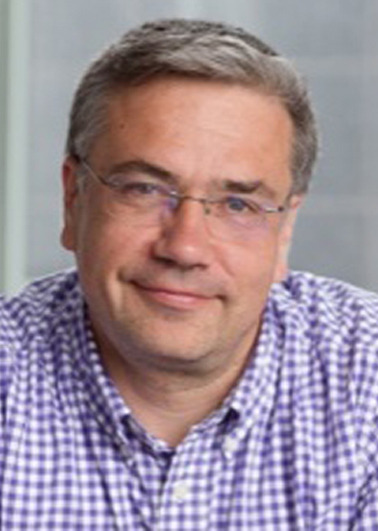# Sustainable and Selective Modern Methods of Noble Metal Recycling

**DOI:** 10.1002/anie.202214453

**Published:** 2022-12-14

**Authors:** Anže Zupanc, Joseph Install, Marjan Jereb, Timo Repo

**Affiliations:** ^1^ Department of Chemistry University of Helsinki P.O. Box 55 (A. I. Virtasen aukio 1) 00014 Helsinki Finland; ^2^ Faculty of Chemistry and Chemical Technology University of Ljubljana Večna pot 113 1000 Ljubljana Slovenia

**Keywords:** Circular Economy, Gold, PGM, Recycling, Silver

## Abstract

Noble metals exhibit broad arrange of applications in industry and several aspects of human life which are becoming more and more prevalent in modern times. Due to their limited sources and constantly and consistently expanding demand, recycling of secondary and waste materials must accompany the traditional mineral extractions. This Minireview covers the most recent solvometallurgical developments in regeneration of Pd, Pt, Rh, Ru, Ir, Os, Ag and Au with emphasis on sustainability and selectivity. Processing—by selective oxidative dissolution, reductive precipitation, solvent extraction, co‐precipitation, membrane transfer and trapping to solid media—of eligible multi‐metal substrates for recycling from waste printed circuit boards to end‐of‐life automotive catalysts are discussed. Outlook for possible future direction for noble metal recycling is proposed with emphasis on sustainable approaches.

## Graphical abstract

Recently developed solvometallurgical methods with the emphasis on green reagents, solvents and strategies are discussed in recycling noble metals Pd, Pt, Rh, Ru, Ir, Os, Ag and Au. Sustainable and separative techniques including selective dissolution, precipitation and coordination for processing of multi‐metal substrates like end of life catalysts and WEEE are highlighted.

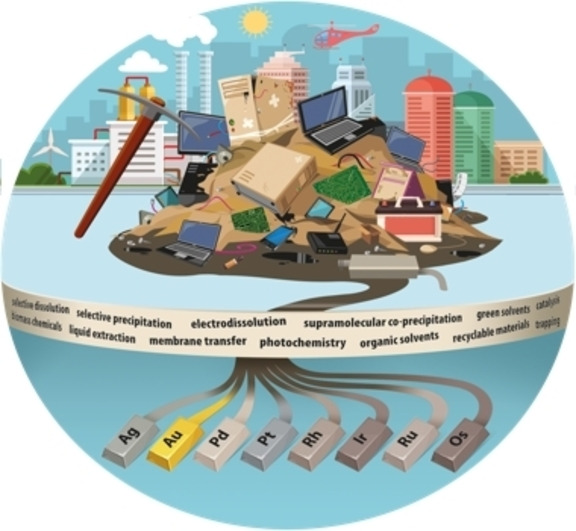

## 1 Introduction

**Figure 1:**
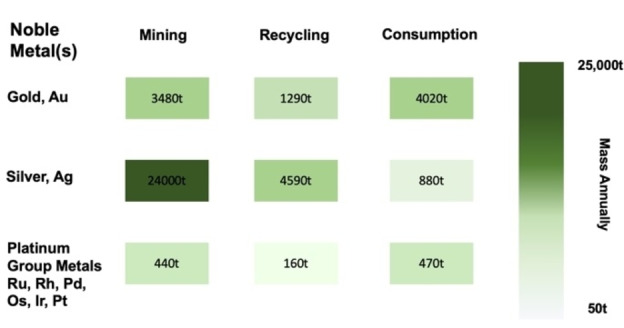
Annual production via mining and recycling of gold, silver and platinum group metals compared to annual consumption.[^3^, ^7^, ^8^]

Lending to their wide array of chemical and physical properties, noble metals of platinum group (or PGMs, mainly Pt, Pd, Rh, Ir, Os, Ru and Ag and Au) have a broad range of applications in several aspects of human life as well as in industry and their demand is expected to grow continuously. The existing mining infrastructure will therefore struggle to match demand, leaving society with an obvious need to recycle from spent materials.[^1^, ^2^, ^3^] (Figure 1) Necessary recycling could also become an economical opportunity—precious metal contents in typical waste electrical and electronic equipment (WEEE), end‐of‐life industrial or automotive catalysts, fuel cells and batteries is significantly higher compared to ores and minerals.^[4]^ Regeneration of critical materials is an important aspect of sustainable development, but utilization of environmentally damaging processes in this regard seems somewhat counterintuitive and calls for application of sustainable approaches in recovery of sparsely available noble metals in the pursuit of “Urban Mining”.[^5^, ^6^]

Metals, metal‐mixtures, and multi‐component substrates that contain non‐metal components like polymers and ceramics can be processed using different techniques as well as combined technologies. Usually, substrates must undergo physical dismantling first. Processes involving delamination, cutting, shredding and grinding is generally followed by particle separation based on magnetic, electrostatic or gravitational methods. Mechanical processing is frequently accompanied by evolution of dust particles, for example, brominated flame retardants found in various WEEE are toxic and bio‐accumulative persistent organic pollutants.

Affordable, yet unsustainable pyrometallurgical processing includes energy intensive roasting and smelting while producing substantial amounts of CO_2_. Alongside release of toxic pollutants and hazardous working conditions, recycling based on thermal treatment usually shows poor selectivity.[^9^, ^10^, ^11^, ^12^] Biometallurgy, that exploits natural mechanisms of organisms or biomolecular interactions with metals, is seen as a green alternative, however due to the slow kinetics, scale‐up challenges and excessive costs, further bio‐leaching strategies need to be developed.[^13^, ^14^] Currently, hydro‐ and/or solvometallurgy, that is based on dissolution in water or other solvents seem like the most advantageous among recycling approaches, providing us with great opportunities for selective separation and sustainable approaches.

Harsh reaction conditions seem unavoidable because of high oxidation potential of these noble metals. Industrially applied traditional (solvo)hydrometallurgical techniques generally depend on oxidative dissolution in strong mineral acids or at highly oxidative conditions which have considerable drawbacks. Sulfuric and hydrochloric acids dissolve most metals while at the same time, explosive H_2_ evolves. Noxious NO_x_ gases are produced when noble metals are dissolved in solutions like corrosive *aqua regia* (1/3 HNO_3_/HCl). Also, toxic oxidants like gaseous chlorine or dangerous mixtures like the *piranha solution* (H_2_SO_4_/H_2_O_2_) are still widely used.

Introducing suitable ligands for complexation to the leaching solution offers opportunity to lower oxidation potentials of noble metals significantly and apply greener oxidants like O_2_ and H_2_O_2_.

Utilized ligands vary from simple halogenide ions to CN^−^ salts in inexpensive but environmentally harmful cyanide leaching. As soft noble metal ions interact better with softer ligands based on sulphur, iodine or even nitrogen, this was exploited to discover more environmentally benign lixiviants. Iodine‐iodide leaching, thiosulfate and thiourea leaching and dissolution techniques based on ammonia present greener dissolution procedures. However, these also come with some disadvantages like low regeneration yield, passivation, or higher price.[^15^, ^16^] These methods are, besides serious health and environmental hazards, accompanied by lack of selectivity of noble metal dissolution. Successful separation of metals with similar oxidation potentials presents a significant unresolved problem. Ideally noble metal recycling should be focused on selectivity as a part of sustainability as well as on the possibilities to apply ecological and economical approaches in other, non‐separative stages.

In solvometallurgical processing choice of solvent is vital and their selectivity and sustainability stem directly from selection. Heavily polluted water streams from hydrometallurgical plants present a great environmental risk. Dissolution and treatment in recyclable organic solvents may alleviate these issues and even enhance selectivity. Dissolution in organic solvents is an approach that gained popularity in recent years[^17^, ^18^] as well as utilization of ionic liquids and deep eutectic fluids.

**Figure 2:**
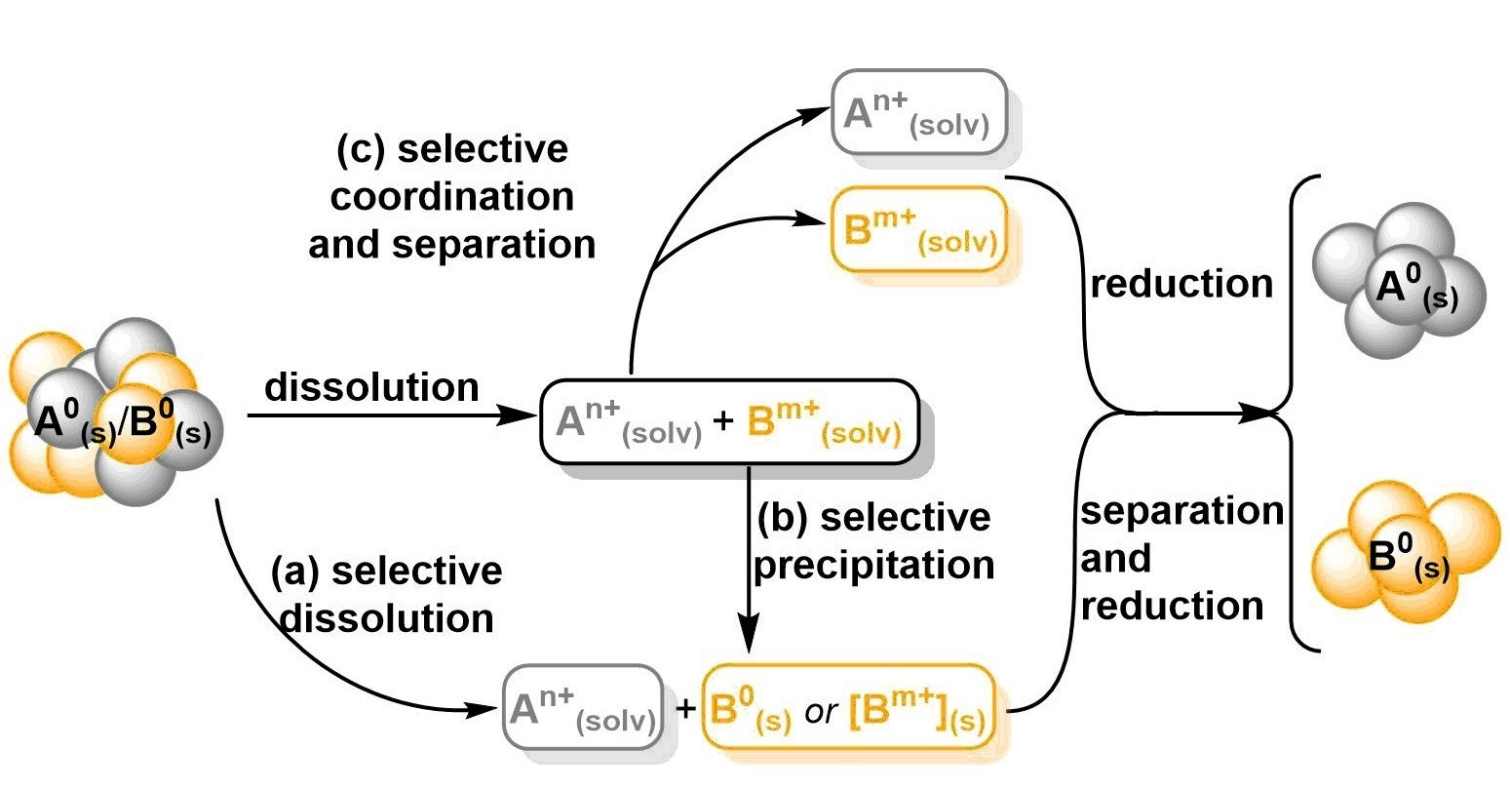
Opportunities for selectivity and sustainability at different stages in solvometallurgical recycling of noble metals. Multi‐metal separation techniques can be roughly divided to (a) selective dissolution, (b) selective precipitation and (c) selective complexation combined with partitioning in different (liquid/liquid or solid/liquid) media.

Selectivity can be introduced through selective dissolution, selective precipitation or selective coordination (Figure 2). Confined by thermodynamics, selective dissolution (a) is dependent on the difference of oxidation potential of metals. This suggests that it mostly relies on the power of the oxidants. However, it can also be adjusted by complexation agents and in cases of Au and Ag it is a vital component of processing.

Selective reductive precipitation (b) is, intuitively the reverse process, and the approach applies. Precipitation of elemental metals can be selective when reductive conditions are carefully chosen—like selective reducing agents or specific potential in electrodeposition. Kinetic control can be utilised if different metals dissolve or precipitate at different rates, separation is then possible with careful control of this process.

Different metal species can also be differentiated based on selective coordination and paired with selective partitioning to different media (c). Selective extraction,^[19]^ membrane transfer,^[20]^ trapping to polymers, solid carriers, frameworks, and materials[^21^, ^22^, ^23^] or even selective (co‐)precipitation of metal salts^[24]^ are all coordinative separation techniques. Clearly, sustainable approaches can be introduced at different stages of hydrometallurgical treatment but utilization of safe, affordable and sustainable ligands, oxidants, reducing agents and other additives are essential for the progression of this field.

Selective oxidation of metals, and dissolution in organic solvents, can be achieved by careful adjustment of oxidation potential with additives and preferential coordination of selected ligands to specific noble metal centers. This enables milder conditions for metal dissolution. Halogens can act as oxidants and together with organic ligands form adducts capable of dissolving noble metals.^[25]^ Thiol‐assisted dissolution can also be performed in the presence of greener oxidants, O_2_ and H_2_O_2_.[^26^, ^27^, ^28^, ^29^] Processing in organic solvents is therefore a great future direction for the selective and sustainable regeneration of these precious and scarce elements and recent developments on the topic are collected in this minireview.

## 2 Platinum Group Metals

Platinum group metals Pt, Pd, Rh, Ir, Os and Ru are important in the chemical industry such as catalysts for ammonia and fine chemical production, petroleum refining, gas remediation and prominently, in automotive industry. They are vital parts of electrolysers and fuel cells and can be—especially Pt—also used in medicinal application and electronics.[^2^, ^3^] The largest share of Pt and majority of produced Pd and Rh are annually spent on manufacture of three‐way car catalysts.[^30^, ^31^] Typical ceramic honeycomb catalyst consists of 0.3–1.0 g kg^−1^ Pt, 0.2–0.8 g kg^−1^ Pd and 0.05–0.1 g kg^−1^ Rh accompanied by cordierite skeleton coated together with small amounts of metal oxides.^[12]^ Less contaminated by base metals as well as hundredfold increase of content compared to typical PGMs ores, these catalysts are examples of incredibly desirable recycling substrates.

Typically, in industry, hydrometallurgical remediation consists of reductive pre‐treatment of passivated oxide surfaces with hydrogen gas that will enhance the PGMs regeneration with concentrated hydrochloric acid. Dissolution ability is heavily dependent on chloride concentration and the presence of toxic Cl_2_ that can be in situ produced electrochemically or by chemical reaction. More expensive iodine‐iodide leaching shows similar efficiency to chlorine‐based dissolution but has not been yet successfully applied on pilot scale.[^32^, ^33^, ^34^, ^35^] For now, recycling of PGMs is sufficiently following their excavation, but demand is expected to expansively grow in the future. New selective remediation techniques focusing on lowering the environmental impact are needed for their continuous and consistently expanding use.

### 2.1 Selective Oxidative Dissolution

**Figure 3:**
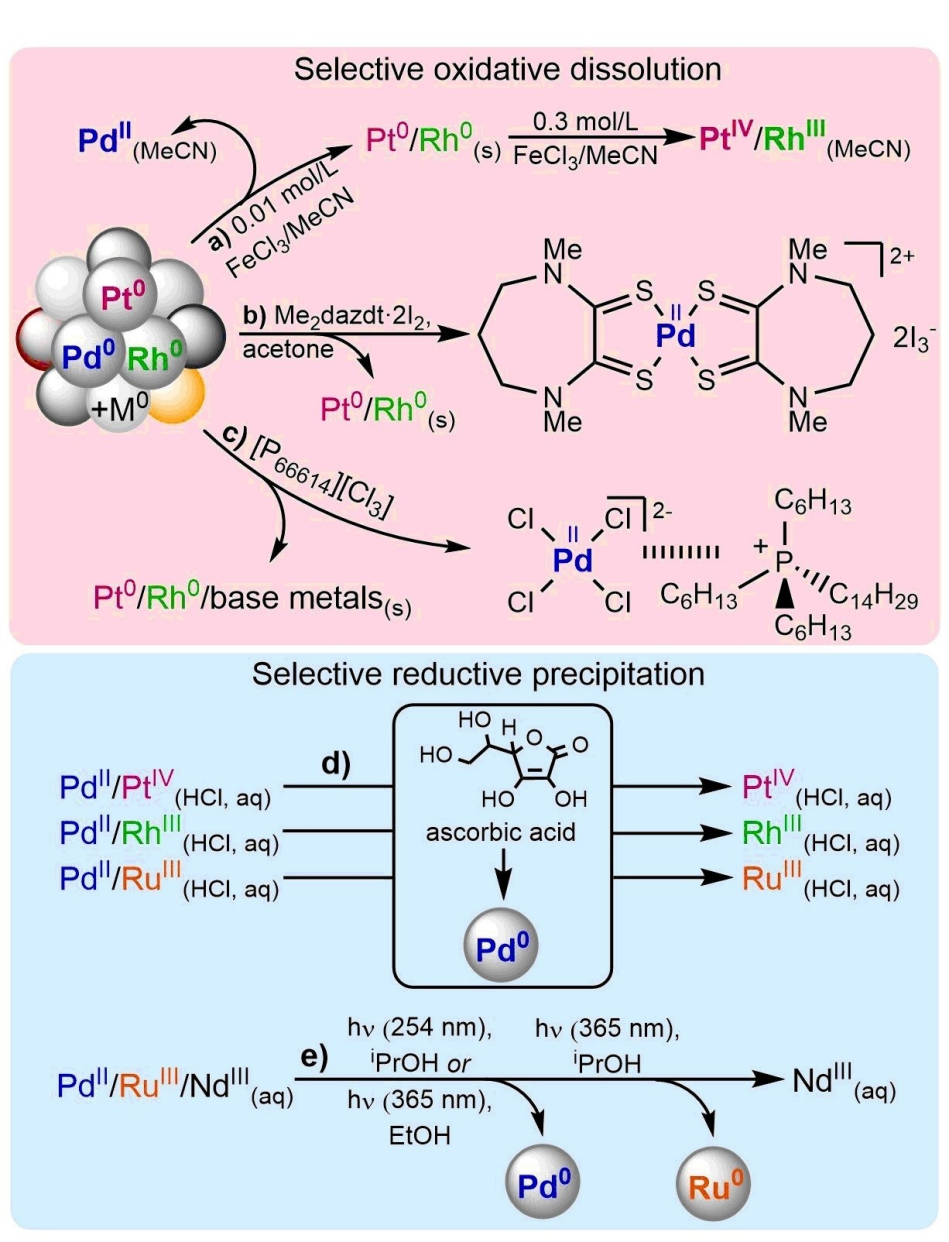
Selective oxidative dissolution of PGM (and base metal) mixtures and selective reductive precipitation from dissolved mixture of PGMs (and other metals).

Although utilization of greener media reduces the environmental impact, they rarely supply the desired selectivity when it comes to PGMs. Their comparable chemistry in non‐aqueous media seems to play the vital role when selective dissolution is intended. Ionic liquids and deep eutectic solvents have been used as leaching systems to dissolve Pt, Pd and even more inert Rh.[^32^, ^33^] Trihalide ionic liquids [P_66614_][Cl_3_] can be even applied for preferential dissolution of Pd from multi‐metal substrate containing base metals, Pt and Rh (Figure 3c).^[34]^

As well as ionic media, dissolution in common organic solvents like acetone, ethyl acetate, acetonitrile, THF and DCM offers an opportunity to efficiently dissolve Pd among other precious metals. Sulphur‐based ligands are prevailing in organic dissolutions, especially when dealing with softer metal ions.[^35^, ^36^]

Thione and dithione based ligands with stoichiometric amounts of halogen oxidant react with metals.[^35^, ^36^, ^37^, ^38^] A unique option to dissolve only Pd in the presence of Pt and Rh is reported when Me_2_dazdt ⋅ 2I_2_ is used as a lixiviant (Figure 3b).[^39^, ^40^, ^41^] Also metal‐ions like Fe^3+^ and Cu^2+^ are capable of oxidizing PGMs in acetonitrile or DMSO and even selective dissolution can be accomplished with careful adjustment of oxidant concentration; diluted FeCl_3_/acetonitrile solution leach Pd first while Pt and Rh require 30‐times higher concentrations of oxidant (Figure 3a).^[42]^ Even with new application of sustainable media going as far as utilization of supercritical CO_2_ ^[43]^ there still remains a distinct possibilities for innovation when selective dissolution of single metal from PGM mixture is the aim.

### 2.2 Selective Reductive Precipitation

Selective reductive precipitation from multi‐ion solution is a somewhat unexploited concept within the scope of PGM recycling. Sparse examples are exploring a well‐known bio‐based reductant, ascorbic acid to—even though incomplete—separation of Pt, Rh, or Ru from Pd in two‐component aqueous HCl solutions (Figure 3d).^[44]^ Considering environmental impact, application of UV‐ or visible‐light driven processes also presents a suitable opportunity.^[45]^ Sequential precipitation of Pd and Ru from lanthanide (Nd) containing waste solutions that are a by‐product of spent nuclear fuel reprocessing can be, for example, realized by photoreduction using sacrificial alcohols (Figure 3e).^[46]^ Induction of selectivity at oxidative dissolution or at the final reduction step seems the most convenient when remediation of single metal from complex multi‐metal substrates route is pursued.

When selectivity cannot be induced at the initial oxidation or the final reduction step, discrimination of metal ions must be done after all metals are dissolved. As shown below, sustainable solvometallurgical methods can be applied for separation of PGMs from base metals and from each other. Selective extraction, membrane transfer, co‐precipitation and trapping to solid media are approaches that rely on difference between ionic PGM species.

### 2.3 Selective Extraction and Membrane Transfer

**Figure 4:**
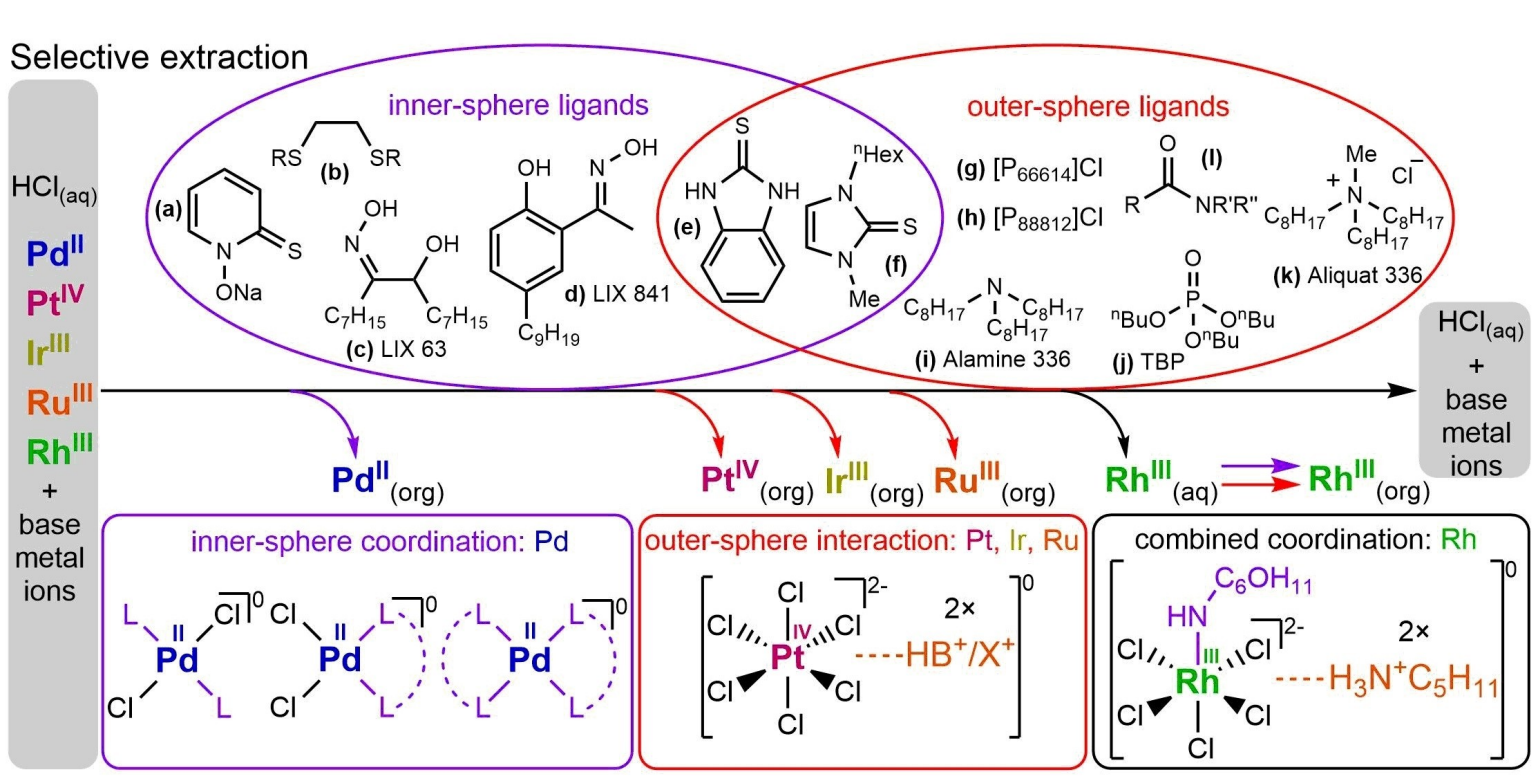
General approaches to separation of PGMs from each other and from base metals by solvent extraction. PGM ions are transported from acidic aqueous solutions to less polar organic solvents by formation of neutral inner‐ or outer‐sphere complexes or to ionic liquids by ion‐exchange reactions.

The most prominent technique for PGMs separation is solvent extraction, primarily from aqueous HCl medium. Different ionic PGM species can be sequentially extracted to more polar ionic media or to less polar organic solvents based on their interaction with ions or substitution reactions on metal centre. Extraction techniques can thus be roughly divided according to type of interaction between metal species and ligand (Figure 4).

Outer sphere coordination between PGM chloride anions and IL (or DES) cations allows [MCl_x_]^(n−x)+^/Cl^−^ anion exchange between two media.[^47^, ^48^] Selective partitioning of Pd, Pt and Rh chloride complexes is possible with combination of phosphonium based ILs like [P_66614_][Cl] and organic solvents like xylene.[^49^, ^50^] [P_88812_][Cl] (Figure 4h) is, on the other hand capable of *p*H‐dependant sequential extraction of Pd^II^ and Rh^III^ from base metals that are present in typical car exhaust catalysts.^[51]^ Proton exchange fuel cells are another important waste substrate. Selective extraction of Pt^IV^ and Co^II^ from leach solutions of carbon deposited Pt_3_Co nanoparticles, is possible with hydrophobic tetradecylpyridinium bis(trifluoromethanesulfonyl)imide ([C_14_py][NTf_2_]).^[52]^ ILs like [P_88812_][Cl] can also be exploited as a part of polymer inclusion membrane. Sequential transport of PtCl_6_^2−^ and PdCl_4_^2−^ is possible and it depends on the receiving solution while leaving [Rh^III^Cl_5_(H_2_O)]^2−^ in the feed solution that emphasizes the importance of specific ion pair interactions.[^53^, ^54^, ^55^] Ion exchange membranes utilising this specific ion pair interaction unlock potential for single‐step selective extraction and examples of Pt,^[56]^ Pd,[^57^, ^58^] and Rh^[59]^ can be found emerging from recent literature. The significance of this method is illustrated in the gold and silver extraction domain which is covered later in this review but there is much potential for manipulations of the ionic interactions on a supported membrane to provide selective extraction of multiple noble metals.

PGMs can also be selectively extracted from aqueous solutions to organic solvents by formation of neutral extractant‐metal complexes which is necessary for efficient phase transfer to more lipophilic environment (Figure 4). Inner sphere complexes with Pd^II^ centre is generally exploited in PGM separation and removal of Pd therefore represents the first step in sequential extraction. Sulphur and oxime‐based ligands—especially chelate type—are capable of substituting chloride anion selectively on Pd^II^ centre while no reaction with other metal chlorides is usually observed. Extractant consisting of dithioethers[^60^, ^61^] (Figure 4b), thioamidated calixarenes,[^62^, ^63^] sodium salt of 2‐mercaptopyridine *N*‐oxide^[64]^ (Figure 4a), amide^[65]^ and diamide^[66]^ ligands and commercially available hydroxy oximes like LIX 63^[67]^ (Figure 4c) and LIX 841^[68]^ (Figure 4d) in chloroform, *n*‐dodecane, toluene or kerosene allow selective extraction of Pd to organic phase, leaving Pt, Rh, Ir, Ru, Cu, lanthanoid, Zn, Fe, Pb, Cr, Mn and other base metal ions in aqueous HCl solution. This is typically followed by sequential extraction of Pt^IV^, Ir^III^ and Ru^III^ chlorides that preferentially interact with outer sphere ligands. TBP (tributyl phosphate)[^67^, ^69^] (Figure 4j), Aliquat 336 (trioctylmethylammonium chloride)^[67]^ (Figure 4k), HCl salt of Alamine 336 (trioctylamine)[^68^, ^69^] (Figure 4i) that are commercially used for separation of PGMs or even simple amide (Figure 4l) and urea type compounds^[70]^ form neutral complexes that are permeable to organic solvents like kerosene and chloroform. At this stage, selectivity is induced by adjusting the concentration of specific ligands. Rh^III^ extraction is challenging and generally presents the last separation step due to the complexity of the Rh^III^ species of consequence from the preceding steps. Combination of inner and outer ligands can be applied for efficient transportation of Rh^III^ to organic phase.[^71^, ^72^, ^73^] Single ligand separation of PGMs, Cu^II^ and base metals are also known with thioureas like 2‐mercaptobenzimidazole^[74]^ (Figure 4e) and 1‐hexyl‐3‐methyimidazole‐2‐thione^[75]^ (Figure 4f). These act as inner sphere ligands for Pt^II^ and outer sphere ligands for Pt^IV^ (or even Rh^III^). Sequential extraction was achieved by adjustment of acidity and/or concentration of ligand in chloroform or in 1‐butanol. Polyethylene glycol‐based nonionic surfactants can be employed in a promising green method know as cloud point extraction to selectively extract PGMs from Cu‐, Ni‐ and Zn‐containing aqueous solutions.^[76]^ Selective solvent extraction is generally followed by stripping or cementation to acquire pure metals or single metal compounds.

### 2.4 Selective Precipitation

Selective precipitation of specific ionic PGM species is an efficient technique for separation. Aqueous HCl solutions that contain Pd^II^ and Pt^IV^ can be, for example treated with NH_4_Cl that forms insoluble (NH_4_)_2_[MCl_6_] salts, but only with PGM in oxidation state of IV. This allows the sequential precipitation and removal of Pt^IV^ compound followed by oxidation of Pd^II^ with NaClO_3_ and precipitation of formed Pd^IV^ salt.^[77]^ Molecular recognition tactic is also viable due to the specific interaction of Pt^IV^Cl_6_^2−^ with cucurbituril forming co‐precipitate while leaving Pd^II^Cl_4_^2−^ and Rh^III^Cl_6_^3−^ in the solution.^[78]^ Even simpler approaches like *p*H adjustment (to 13) can be applied for specific Pt and Ru containing solutions that results in Ru‐hydroxide precipitation and therefore addresses the separation problem.^[79]^

### 2.5 Selective Trapping

PGMs can be trapped selectively to solid media that usually consists of inorganic (SiO_2_, dichalcogenides), metal–organic framework and polymeric and biomass derived[^80^, ^81^, ^82^, ^83^, ^84^, ^85^] (Table 1, entries 1–6) solid substrate with (tethered) ligand moieties. Functionalities are designed to specifically bind to one or more PGM species and absorb them from the multi‐metal containing aqueous solutions. Various mechanisms can be exploited relying on reactivity of specific PGM. Pd^II^ can for example form an inner sphere complex with S‐ and N‐ based tethered ligands[^86^, ^87^, ^88^] or interact with ionic moieties[^89^, ^90^] to remove it from other PGMs and/or base metals (entries 7–10) whereas different metal‐trap interactions have to be exploited to efficiently remove both anionic and cationic Rh^III^ aqua/chloride species from acidic aqueous solutions^[91]^ (entry 11). Pt^IV^ can be efficiently separated from Co^II^ using ion exchange resins while processing the beforementioned proton exchange membrane fuel cells^[92]^ (entry 12) while specific metal‐metal interactions can be exploited to remove Pt^II^ from other PGMs and base metals^[93]^ (entry 13). What is more, complete separation of Pd, Pt, Rh, Ir and base metals is possible by utilization of various commercially available SuperLig systems and careful adjustment of redox potential^[94]^ (entry 14). Trapping to various substrates is usually followed by stripping of the desired PGM ion, even though selectivity based on different retention times can also be realized^[95]^ (entry 15).

**Table 1:** Selective trapping of PGM ions from multi‐metal solutions.

Entry	Trapped Ions	Substrate Solution	Solid Substrate	Functionalization/Active Moiety	Ref.
**1**	Sequential trapping of Au^III^, Pd^II^ and Pt^IV^	Acidic aqueous solution containing Te^IV^, Ga^III^, Gd^III^, Pb^II^ and Cu^II^	Functionalized cellulose	Carboxylate and amino/ammonium groups	[80]
**2**	Pd^II^ and Pt^IV^	Acidic aqueous solution	Functionalized chitosan	1,10‐phenanthroline or 2,2′‐bipyridine motifs	[81]
**3**	Pd^II^	Acidic aqueous solution containing Co^II^, Ni^II^, Zn^II^, Pb^II^ and Fe^II^	Functionalized silica nanoparticles	Proline‐valinol amide motif	[82]
**4**	Au^III^, Pd^II^, Pt^II^, Ag^I^ or Ru^III^	Aqueous solutions containing interferences like Cu^II^, Ni^II^ and Al^III^	MoS_2_ or WS_2_ transition metal dichalcogenide paper	MoS_2_ or WS_2_	[83]
**5**	Pd^II^ and Au^III^	Aqueous solutions	MOF based on: 3‐formyl‐4‐hydroxybenzoic acid, 2,6‐diaminopyridine and ZrCl_4_	N‐ and O‐containing functional groups	[84]
**6**	Au^III^, Pt^II^ and Pd^II^	Acidic aqueous solution containing Ni^II^, Cu^II^ and Zn^II^	Starbon® (starch‐derived carbonaceous mesoporous material)	O‐containing functional groups	[85]
**7**	Pd^II^	Acidic aqueous solution containing Pt^IV^, Rh^III^, Zr^IV^, Ce^III^, Ba^II^, Al^III^, La^III^ and Y^III^	Functionalized Amberlite XAD‐7	Thiocarbamoylated calixarene motif	[86]
**8**	Pd^II^	Aqueous solution containing Zn^II^, Cd^II^, Fe^II^, Pb^II^ and Ni^II^	Functionalized porous organic polymer	*o*‐aminopyridine moiety	[87]
**9**	Pd^II^ and Pt^IV^	Acidic aqueous solution containing Rh, Fe, Zn, Ce, Pb, Mn, Sr, Cu, Ni, Cr, B, Mg, La, Ti and Mo ions	Silica gel functionalized with organic polymer and ionic liquid	Phosphonium chloride ionic liquid motif	[99]
**10**	Pd^II^	Acidic aqueous solution containing Cu^II^, Fe^II^, Zn^II^, Ni^II^ and Cr^VI^	Mesoporous silica composite with ionic liquid	Phosphonium chloride ionic liquid	[90]
**11**	Rh^III^	Acidic aqueous solution	1,3,5‐Triazine‐pentaethylenehexamine resin	Amino groups	[91]
**12**	Pt^IV^ and Co^II^ sorption followed by sequential desorption	Acidic aqueous solution	Lewatit‐MP62 ion exchange resin	Tetraalkylammonium chloride moiety	[92]
**13**	Pt^II^	Aqueous solution containing Pt^IV^, Pd^II^, Au^III^,, Ag^I^, Cu^II^ and Ni^II^	2D supramolecular polymer	Pt^II^ complex	[93]
**14**	Sequential sorption of Pd^II^, Pt^IV^, Rh^III^ and Ir^III^	Acidic aqueous solution containing base metals	Functionalized silica gel	Tethered crown ether with different sizes	[94]
**15**	Sequential elution of Pd^II^, Au^III^ and Pt^IV^	Aqueous solution containing Na^I^, K^I^, Ba^II^, Mg^II^, Ca^II^, Ni^II^, Sr^II^, Co^II^, Cu^II^, Pb^II^, Fe^III^ and Al^III^	Functionalized silica gel	Different tethered macrocycles	[95]

### 2.6 Osmium recycling

Of the noble metals recovery methods mentioned osmium is sparsely included, mostly due to its limited use in the dominant substrates, car exhaust catalysts. However, this doesn't neglect the importance of recycling methods for osmium. The numbers of industrial uses may be few, as catalysts in the chemical industry and tissue straining for microscopic methods are the most distinct, however due to its rarity and crucially, toxicity in it's tetroxide form, recovery to the elemental metal is required.^[96]^ From OsO_4_ to regenerate osmium metal it requires reduction, this had been achieved using biomass derived 10‐undecenoic acid in combination with a polymer support.^[97]^ Osmium is also present in the lead sludge obtained from copper production at 50 g t^−1^, in addition to 800 g t^−1^ of rhenium.^[98]^ This can be extracted on the first stage of copper processing providing a promising source of these rare noble metals.

## 3 Gold and Silver

Within the various substrates, gold and silver can constitute a substantial portion of the wt % of precious metals. A descriptive example of this, besides ubiquitous waste cell phones, is in printed circuit boards (PCBs), gold and silver in some cases amount up to 3.6 g kg^−1^ and 12.3 g kg^−1^, respectively, compared to small content of palladium around 0.1 g kg^−1^.^[100]^ As aforementioned, pyrometallurgical techniques contain substantial drawbacks for selectivity and complications from ceramic components in substrates as well as a large expense of energy. For this reason, in this section the focus will be on modern hydrometallurgical processes for the extraction of gold and silver.

### 3.1 Selective Oxidative Dissolution

Once a substrate has been successfully separated into the metallic and non‐metallic components via physical separation methods, it is typically dissolved in a leaching solution followed by selective extraction, precipitation, ion‐exchange membranes or cementation. Cyanide leaching despite its risks for industrial hazards, is the most commonly employed lixiviant.^[101]^ Another distinct drawback to this approach is the strong solubility of copper in cyanide and tied with the large amounts of copper present in the substrates used it presents a large problem. An approach to negate this is a multi‐step leaching method in which extraction of base metals such as copper can be removed before applying the final leaching steps.^[102]^

Alternatives to cyanide leaching have been extensively studied and two prevalent alternatives are thiourea and thiosulfate, however both with their own distinct disadvantages. Thiourea provides fast leaching in acidic loads and compatible with small quantities of additional metals^[103]^ (copper and iron) however it suffers from fast degradation and lacks tolerance for basic materials, restricting the substrates used for extraction.^[104]^

**Figure 5:**
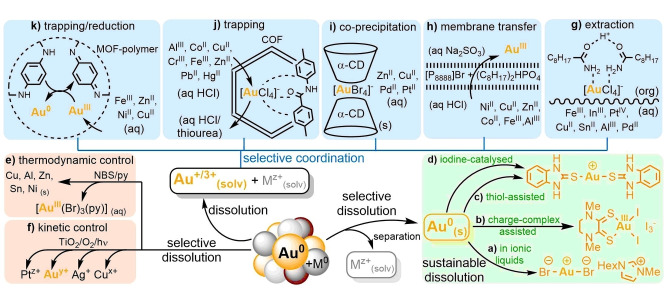
Selectivity and possibilities of sustainable approaches in solvometallurgical gold recycling. Gold can be dissolved in novel media (a–d) after metals with lower oxidations potential are removed first. Preferential dissolution (e) and time‐dependent dissolution (f) are also applicable. Separation of multi‐metal solutions can be achieved by selective coordination (g–k).

Organic media can be applied for selective and sustainable dissolution. From moderately toxic halogens in alcohols^[17]^ or noxious *organic aqua regia* ^[105]^ to more sustainable systems using recyclable PPh_3_Cl_2_ or oxalyl chloride in the presence of H_2_O_2_ can be employed to dissolve Au and other noble metals in acetonitrile.^[106]^ Less corrosive and volatile system based on halogens trapped as X_3_^−^ in ionic liquids[^107^, ^108^] (Figure 5a) or halogens in deep eutectic solvents like ethaline^[109]^ are also known. Ligand based dissolution in organic solvents has also been extensively studied. Charge complexes between a sulphur atom bearing ligands and halogens can efficiently transform gold to well‐defined compounds (b).[^110^, ^111^, ^112^, ^113^] Even in the presence of more sustainable oxidants like O_2_ or H_2_O_2,_ thiones can act as stabilizing ligands for the dissolved gold ions.[^26^, ^27^] Ligand exchange reactions on liable Au^I^ centre allow sustainable approaches based on two ligands. The stoichiometric amount of 4‐mercaptopyridine is, for example, capable of transporting gold ions into the DMF solution where it is replaced with much cheaper 2‐mercaptobenzimidazole that acts as stabilizing agent (c).^[29]^ Active species for similar approach in ethanol can also be catalytic amount of iodine that is regenerated by H_2_O_2_ when iodide is expelled from the [AuI_2_]^−^ species (d).^[28]^ Catalytic reactions and green solvents as well as solvent‐free transformations^[112]^ are all objectives of sustainable development.

There also exists excellent green alternatives such as glucose and modified chitosan resins for silver recycling.[^106^, ^114^] The silver in its +1‐oxidation state can coordinate to various sites within these carbohydrate derivatives and will be reduced and precipitate as a silver nanoparticles. Such green lixiviants are highly promising for further extractions of silver and other noble metals.

Ideally, dissolution of multi‐metal mixture could be relatively selective for gold as in the example of NBS‐pyridine combination in water^[115]^ (Figure 5e) relying on thermodynamics. Alternatively, exploiting kinetic control seems a very promising approach. For example, in organic solvents^[116]^ or water^[117]^ based TiO_2_‐photocatalytic system, which results in different metals selectively dissolving at different times (f).

### 3.2 Selective Extraction and Membrane Transfer

A far more common tactic in (solvo)hydrometallurgical gold processing is coordinative separation from multi‐metal solutions. Size, charge and shape driven differentiation of ions as well as highly specific supramolecular interactions offer great selectivity as well as lesser environmental impact when greener compounds and systems are used. Selective transfer of [AuCl_4_]^−^ is possible by aqueous‐to‐ionic liquid,^[118]^ aqueous‐to‐surfactant phase by cloud point extraction^[119]^ or by aqueous‐to‐organic solvent extraction with simple amides[^120^, ^121^] (Figure 5g) or phosphines.[^106^, ^118^] Further to this with green and renewable diethyl carbonate, a more sustainable alternative to frequently used methyl isobutyl ketone and dibutyl carbitol that is capable of selectively transport of Au^III^ over Cu^II^ from important secondary source—copper anode slime—solution. Aqueous‐to‐aqueous media transfer through polymer inclusion membrane is also possible with amic acid based extractants.^[122]^ Even hydrophobic deep eutectic solvents^[123]^ (h) can also be applied in this manner as well as micellar‐enhanced ultrafiltration that allows separation of AuCl_4_^−^ and PdCl_4_^2−^ based on their difference in the interaction with amphiphiles and chelating agents.^[124]^ Particularly in the domain of silver, but also important in gold extractions, ion exchange membranes are a dominant pursuit in selective and efficient recovery from leachate solutions.

**Figure 6:**
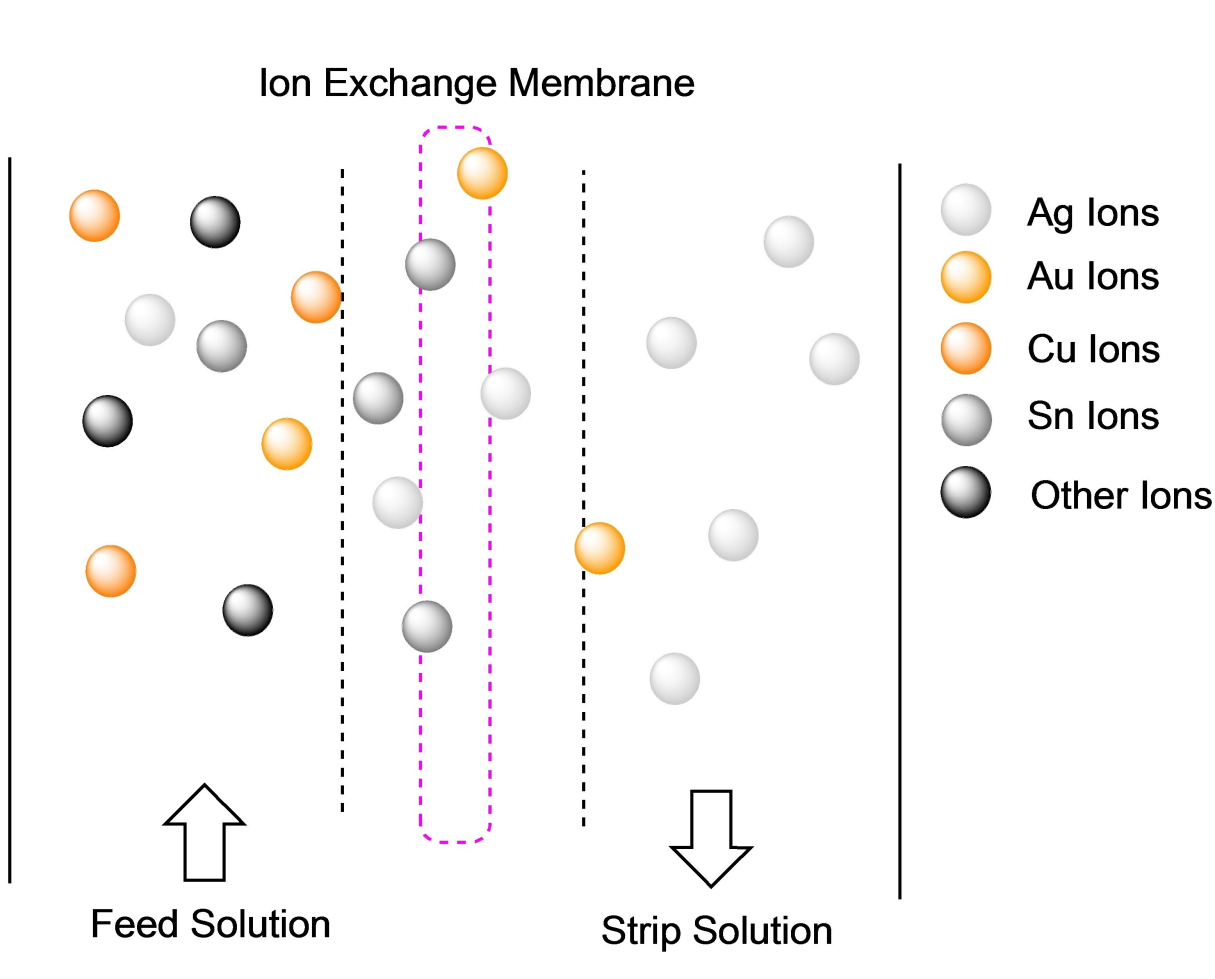
Ion exchange membranes selective separation of specified metal ions, Ag and Au.

As depicted in Figure 6, a leachate solution, acid, base or thiourea dependant on the system, are flowed pass an exchange surface where transfer of ions occurs into the strip solution of which can be fed for subsequent purification. Examples of ion exchange resins include highly efficient recovery of Au and Ag from base metal‐chloride^[125]^ and thiosulphate^[126]^ feed solutions in a simple, single step procedure.

### 3.3 Selective Precipitation, Trapping and Reduction

Selective co‐precipitation of gold ions from multi‐metal solution can be achieved by supramolecular interaction strategy promptly using cucurbiturils^[127]^ and cyclodextrins (i).^[128]^ α‐Cyclodextrin is even capable of preferential stripping of [Au(CN)_2_]^−^ providing a well needed Au/Ag separation after adsorption to activated carbon from cyanide liquor.^[129]^ With emphasis on regeneration and renewable materials, well‐defined precipitated co‐crystals can be formed selectively by simple and recyclable diamide^[130]^ or natural compounds like niacin.^[131]^

Selective trapping of gold ions to polymers or solid supported ligands based on natural compounds like glycine,^[132]^ methionine^[133]^ and porphyrin^[134]^ and overall, compounds based on abundant H, C, N, O and S elements seems like another prospect. Different porous framework materials have been applied for selective adsorption—amide‐linked covalent organic framework (COF) JNU‐1 exhibits fast kinetics and possibility of selective adsorption and desorption depending on the feed or extractive solution (j).^[135]^ Stripping of the adsorbed ions as well as reduction to elemental gold are important parameters when it comes to trapping. Regenerable MOFs, redox‐active poly(para‐phenylenediamine) composite (k) and thiourea‐resorcinol‐formaldehyde microspheres^[136]^ can for example selectively trap and reduce gold ions.^[137]^ What is more, Mn^II^‐doped MoS_2_ can photocatalytically reduce gold ions from thiosulfate solution^[138]^ which is more atom efficient and therefore more environmentally friendly than industrially applied Mg or Zn cementation. Taking advantage of gold's lower reduction potential, selective precipitation from multi‐metal solutions can also be achieved by biomass‐derived compounds like oxalic acid^[139]^ as well as by electrowinning that sometimes comes with issues in efficiency and purity of end‐product. Research on electrodeposition has been done, for example on replacing cyanide with more sustainable compounds like methionine^[140]^ and on promising techniques like electrodeposition‐redox replacement.^[141]^ Even when extra steps are considered in separation of multi‐metal solutions, high selectivity and associated sustainability often justify their use.

## 4 Summary and Outlook

This Minireview gives an overview of the recent, emerging methods to sustainable noble metal recycling. Noble metals are extensively used in consumer and commercial electronics, chemical and automotive industry, production of batteries, fuel cells, in sensing, medicine as well as in non‐industrial applications. Broad array of applications emerges from their desirable properties like oxidative stress resistance combined with conductivity and catalytic activity. Combination of ‘noble’ features, mainly high oxidation potential as well as high melting point does not only make them highly exploitable, but at the same time, hard to reprocess and recycle. With their low abundance and ever‐growing demand, economically available mineral sources of noble metals, particularly PGMs (Pd, Pt, Rh, Ru, Ir, Os), Ag and Au are rapidly beginning to outweigh supply. Recycling of secondary and out‐of‐use materials promptly from urban waste streams is essential.

Traditionally applied technologies depend on energy‐intensive pyrometallurgical processing or, on the other hand, utilization of highly oxidative and acidic conditions in hydro‐ and solvometallurgical processing that includes strong acids like HCl, H_2_SO_4_, HNO_3_ and dangerous *aqua regia* and *piranha solution*, toxic oxidants like Cl_2_ or even use of cyanide and mercury, that possibly produce big amounts of toxic and/or corrosive waste. Utilization of ‘hard’ conditions also attributes to low selectivity and that adds to the environmental impact. This goes against objectives of sustainable development, which seem somewhat counterintuitive when recycling, a principle of circular economy and green chemistry is at aim.

Waste PCBs and discarded mobile phones that contain Au, Ag, Pd and Pt as well as Cu, Fe and other base metals, automotive catalysts with significant amounts of Pd, Pt and Rh, proton exchange fuel cells with big content of Pt next to Co, Ag‐rich silver batteries, plant wastewaters and others, all present difficult substrates due to their complex composition. Within the confines of solvometallurgical recycling, physical processing of waste substrates is usually followed by oxidative dissolution step.

Modern methods based on dissolution in organic solvents are likely to play a significant role. Lending to the variety of compounds soluble in organic media, more diverse chemistry can be applied to solve the separation problem as well as to address the sustainability by promptly replacing halogenated and hydrocarbon solvents for green alcoholic media. Safe, inexpensive, and environmentally benign solvents, oxidants, reductants, ligands, additives, solid traps and carriers are desired to improve the sustainability of processes. Processes that do not produce large amounts of waste including catalytic and photochemical systems that quantitatively proceed under mild conditions are preferred. With not yet many existing examples, design of highly selective, ligand‐dependent systems that induce the selectivity in minimal steps seem highly attractive future directions for inevitable recycling of noble metals.

## Conflict of interest

The authors declare no conflict of interest.

## Biographical Information

*Anže Zupanc studied at Faculty of Chemistry and Chemical Technology, University of Ljubljana in Slovenia where he received his Ph.D. (2022) under the supervision of Prof. Marjan Jereb. He is currently working at the same faculty and will soon join prof. Timo Repo's research group at University of Helsinki as a postdoctoral fellow. His research interest includes green transformations of halogenated and chalcogenated organic compounds and noble metal recycling*.

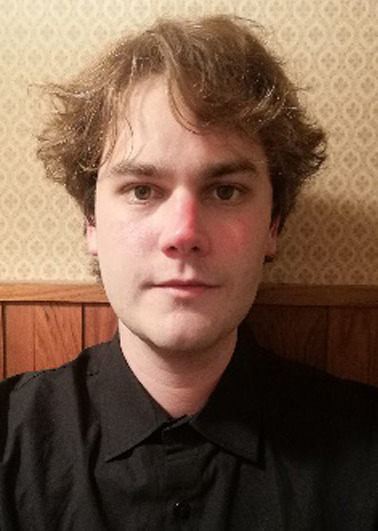

## Biographical Information

*Marjan Jereb is Professor at the Faculty of Chemistry and Chemical Technology at the University of Ljubljana, Slovenia. He obtained his Ph.D. from University of Ljubljana in 2001 and done a post‐doctoral stay with Professor Antonio Togni at ETH Zurich. His main research interests focus on green organic chemistry, including research of organohalogen and organochalcogen chemistry*.

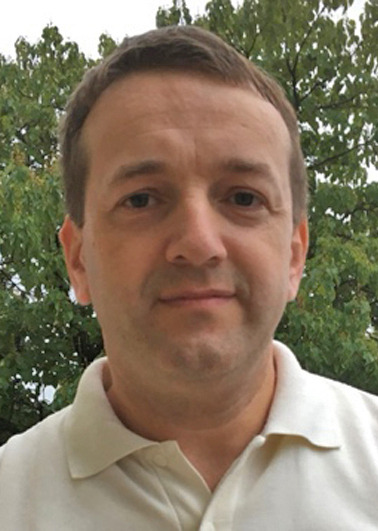

## Biographical Information

*Joseph Install completed his undergraduate studies at the Department of Chemistry at The University of York (UK) before moving to the University of Helsinki, Finland to begin Ph.D. studies under the supervision of Professor Timo Repo in 2021. His research interests are green and sustainable approaches to modern chemistry for a circular economy, including green process design*.

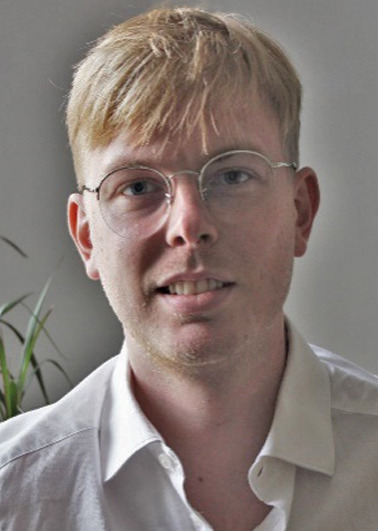

## Biographical Information

*Timo Repo received his Ph.D. in 1997 from University of Helsinki (Finland), where he was nominated as a full professor of inorganic chemistry in 2007. His research is focused on green chemistry and homogeneous catalysis, including catalyst development for activation of small molecules (e.g. O_2_, CO_2_, H_2_), oxidation, reduction, C‐H activation, and biomass valorisation*.